# Research and Remedies

**Published:** 1911-11-04

**Authors:** 


### RESEARCH AND REMEDIES.

### Cancer Research.

The tenth volume of the Annual Cancer Research Report from the Middlesex Hospital shows two points very fully. These are the vastness of the subject and the very small knowledge which so far has been gleaned concerning it. In this report are numerous painstaking papers dealing with researches of greater or less complexity and laboriousness. No one of these researches can honestly be asserted to carry us one single step nearer the solution of the cancer problem, though some of them may very possibly do so indirectly by providing the basis of assured knowledge which shall enable some future worker to progress along lines not yet thought out. The most interesting contribution to this issue is, however, that of the Director of the Laboratories, who summarises the most important results of the work done since his appointment to the Directorship. The whole of Dr. Lazarus-Barlow's survey is interesting, and may be recommended to those who take a special interest in the cancer problem.

### A Hospital "Experiment."

A most apt illustration of the remarks made last week in these columns on the subject of so-called "experiments" in hospitals (The Hospital, Oct. 28, p. 106) was furnished on the very day that those remarks were published. For on Friday, Oct. 27, a demonstration was given at University College Hospital of the new method of anæsthesia by venous transfusion, originated last year in Germany by Burckhardt. This interesting development has been introduced into England by Dr. F. S. Rood, and a description of the technical details of the administration appeared recently in the *British Medical Journal*. Without entering into particulars, the general principle is simple enough. A five per cent. solution of ether in (warm) normal saline is slowly run into one of the superficial veins of the arm, through a cannula of the same type as is used for the familiar method of saline transfusion. To minimise the discomfort of this proceeding local anæsthesia is used, and the arm is lightly fixed to a wooden splint to avoid the undesirable factor of reflex or purposive movement.

The advantages claimed for this strikingly novel method of administering ether are such that for certain classes of operation its further trial is a necessity. If it turns out that corresponding disadvantages, such as the risk of thrombosis and embolism, are absent or negligible, it is certain that a real advance has been made which will render surgical work much easier of performance in these special cases. The operations in question include cleft palate, removal of the jaw, excision of the tongue, the larynx, dissection of the floor of the mouth and of the neck, and similar procedures; tracheotomy in particular will be robbed of half its terrors. It is easy to see the reason. In the operations named the anæsthetist and the surgeon have to share the field of operation, and each interferes with the other. Moreover, the anæsthetist must preserve a light anæsthesia, for fear of post-operative complications from pus, blood, or mucus accumulating in the air passages; while the surgeon wants as deep a narcosis as possible so that the parts may be little disturbed by the respiratory excursions. The interests of the two are therefore antagonistic. With this new method the greater part of this difficulty disappears. Anæsthesia is said to be easily induced and maintained with much smaller quantities of ether than when it is offered for inhalation; and the supply can be cut off at a moment's notice when the operation is over, or should signs of overdose be manifest. Moreover, the saline infusion in which the ether is given is itself of value in preventing shock. In calling attention to this important matter, it is well to emphasise the fact that the suffering poor in our hospitals have had the benefit of venous anæsthesia before anyone else in this country. On the occasion in question at University College Hospital a man was anæsthetised for excision of the upper jaw; and the "experiment" was highly successful. It may be trusted that other hospitals will give the method a trial; for the apparatus required is simple and inexpensive.

### Burns of the Eye

The treatment of burns of the eye, whether caused by flying sparks and particles, caustics, steam, or boiling liquids, is still in a stage of development, and results are still often unsatisfactory. In the *Prescriber*, Dr. P. A. Harry advocates the use of chloretone dissolved in liquid paraffin, which considerably reduces the percentage of serious after-effects from these disastrous accidents. A saturated solution is used, and is dropped in from an ordinary eye-drop bottle. On admission of such a case, regardless of the source of the injury, the face and external surface of the lids are cleansed with green soap and water. The conjunctival sac is douched with normal saline until all foreign particles are removed. It may be necessary to use a fine pair of forceps to detach small pieces of coal or metal that have become adherent to the conjunctiva. The everted lids must be carefully searched for such particles. In the case of the cornea a spud may be used, though it is not advisable to separate the opaque coagulated layer from the corneal surface. For the first twelve hours an ice-pad is applied to the injured eye. If there is much œdema of the lids the outer canthus must be slit for half an inch. During this period picric acid solution (10 grains to the ounce) is instilled hourly. After twelve hours chloretone in paraffin is substituted, two or three drops being used every half-hour. The eye is now left without pad or bandage, and the patient instructed to move it about as much as possible. Symblepharon is thus reduced to a minimum. The use of a probe to prevent this complication is strongly condemned by the author.

